# Re-analysis with cage as the experimental unit: effect of feeding biochar, oat hulls, yeast fermentate, and organic acids on reduction of *Campylobacter* in free-range broilers from hatching to slaughter

**DOI:** 10.1016/j.psj.2026.107912

**Published:** 2026-10-11

**Authors:** Nao Takeuchi-Storm, Marta M. Dolcet-Negre, Cristina Calvo-Fernandez, Annette Nygaard Jensen, Giulia Ravenni, Marianne Sandberg, Ulrik Birk Henriksen, Brian Lassen

**Affiliations:** a DTU National Food Institute, Research Group for Food Microbiology and Hygiene, Henrik Dams Allé, 2800 Kgs., Lyngby, Denmark; b Faculty of Sciences, Department of Statistics, University of Salamanca, Plaza de los Caídos s/n, Salamanca, 37008, Spain; c DTU National Food Institute, Research Group for Foodborne Pathogens and Epidemiology, Henrik Dams Allé, 2800 Kgs., Lyngby, Denmark; d DTU Chemical Engineering, Department of Chemical and Biochemical Engineering, Frederiksborgvej 399 P.O.B. 49, Roskilde, 4000, Denmark

**Keywords:** Experimental unit, Pseudoreplication, Mixed-effects model, Broiler, *Campylobacter*

Dear Editor,

We write regarding our article “Effect of feeding biochar, oat hulls, yeast fermentate, and organic acids on reduction of *Campylobacter* in free-range broilers from hatching to slaughter” (Takeuchi-Storm et al., 2025). In the study, five treatment groups were housed in separate cages, and the trial was repeated over three rotations: 15 cages and 411 sampled broilers. The published analysis reported significantly lower *C. jejuni* concentrations than the control for biochar-supplemented feed on day 59 and for organic acids in drinking water on day 36.

After publication, we revisited the analysis following discussion of the appropriate experimental unit. Because diets were allocated to cages rather than individual birds, and birds within the same cage shared feed and water, observations from birds in the same cage were not strictly independent. We therefore re-analysed the data using cage as the experimental unit, to account for this dependency.

For this re-analysis, we used a mixed-effects model of log_10_ CFU/g of *C. jejuni*, with group and rotation as fixed effects and cage as a random effect (*lmer*, with Satterthwaite degrees of freedom; Bates et al., 2015; Kuznetsova et al., 2017). Each slaughter day was analysed separately, and the four contrasts against the control within a day were adjusted by the Benjamini-Hochberg procedure. The residual degrees of freedom reduced from 184 to 197 to approximately 8. Consequently, the confidence intervals widened and none of the treatment effects remained statistically significant (Table 1).

**Table 1:** Contrasts between each intervention group and the control, from mixed-effects models with cage as a random effect. Estimates are differences in log10 CFU/g of *C. jejuni* in cecal content.

Day	Comparison	Estimate		95% CI	df	P	P (BH)
36	Biochar - Control	0.59	−0.95 to 2.13		8.0	0.254	0.379
36	Oat hulls - Control	0.42	−1.13 to 1.96		7.9	0.410	0.410
36	Yeast fermentate - Control	−0.55	−2.09 to 0.99		8.0	0.284	0.379
36	Organic acids - Control	−0.59	−2.14 to 0.95		8.0	0.252	0.379
59	Biochar - Control	−0.72	−1.84 to 0.40		8.0	0.074	0.294
59	Oat hulls - Control	−0.26	−1.39 to 0.86		8.2	0.473	0.837
59	Yeast fermentate - Control	−0.09	−1.22 to 1.03		8.0	0.793	0.837
59	Organic acids - Control	−0.07	−1.20 to 1.05		8.0	0.837	0.837

CI, confidence interval; df, Satterthwaite denominator degrees of freedom; P, unadjusted P-value; P (BH), Benjamini-Hochberg adjusted P-value within day.

Treating non-independent measurements as independent observations is commonly referred to as “pseudoreplication” (Hurlbert, 1984). It inflates the apparent sample size and degrees of freedom, narrowing confidence intervals and lowering P-values, so effects may be declared significant.

The observed direction or magnitude of the estimated treatment differences are essentially unchanged; what changes is the statistical uncertainty around them. This does not alter our overall interpretation of the study: further experiments are needed to assess the potential effect of the tested interventions.

For future feeding trials, increasing the number of cages per treatment group, rather than the number of birds within cages, is what improves the power to detect treatment effects.

We bring this re-analysis to readers’ attention in the interest of transparency, and to support the interpretation and design of future intervention studies.

## Disclosures

The authors declare that they have no known competing financial interests or personal relationships that could have appeared to influence the work reported in this paper.

## Author Contributions

Nao Takeuchi-Storm and Annette Nygaard Jensen conceived the idea for the re-analysis. Marta M. Dolcet-Negre performed the statistical analyses. Nao Takeuchi-Storm, Annette Nygaard Jensen, and Marta M. Dolcet-Negre interpreted the results and drafted the initial manuscript. Cristina Calvo-Fernandez, Giulia Ravenni, Marianne Sandberg, Ulrik Birk Henriksen, and Brian Lassen contributed to the interpretation and discussion of the findings and critically revised the manuscript for important intellectual content. All authors reviewed and approved the final version of the manuscript and accept responsibility and accountability for all aspects of the work, ensuring that any questions related to the accuracy or integrity of any part of the work are appropriately investigated and resolved.
